# Bilateral nevus comedonicus of the eyelids: An unusual cause of ptosis and ectropion

**DOI:** 10.1016/j.ajoc.2019.100579

**Published:** 2019-12-17

**Authors:** T. Adam, R. Hage, C. Ahomadegbe, V. Molinié, E. Baubion, H. Merle

**Affiliations:** a Department of Ophthalmology, Centre Hospitalier Universitaire de Martinique, Martinique, French West Indies, France; b Department of Anatomy and Pathological Cytology Dermatology, Centre Hospitalier Universitaire de Martinique, Martinique, French West Indies, France; c Department of Dermatology, Centre Hospitalier Universitaire de Martinique, Martinique, French West Indies, France

**Keywords:** Nevus comedonicus, Ptosis, Ectropion

## Abstract

**Purpose:**

Nevus comedonicus is a rare developmental abnormality of the infundibulum of the hair follicle.

**Observation:**

We report here an unusual case of bilateral extensive nevus comedonicus of the eyelids complicated by bilateral ptosis and ectropion of the lower eyelids. Blepharoplasty was performed on both upper eyelids. Histopathological findings on skin biopsy typically show large, grouped, dilated follicular ostia filled with keratin.

**Conclusions and importance:**

This case is unusual as regards the late-onset (lesions first appeared at age 35) and location of the nevus comedonicus on both eyelids.

## 1 Introduction

Nevus comedonicus is a rare developmental abnormality of the infundibulum of the hair follicle characterized by an aggregation of dilated follicular orifices filled with keratinous material. The disorder is usually an isolated manifestation, but has been associated with skeletal defects, abnormalities of the central nervous system and ocular lesions to constitute nevus comedonicus syndrome.^1^ Diagnosis is made chiefly on clinical grounds and confirmed by histology. We report here an unusual case of bilateral extensive nevus comedonicus of the eyelids complicated by bilateral ptosis and ectropion.

## 2 Case report

**Fig. 1:**
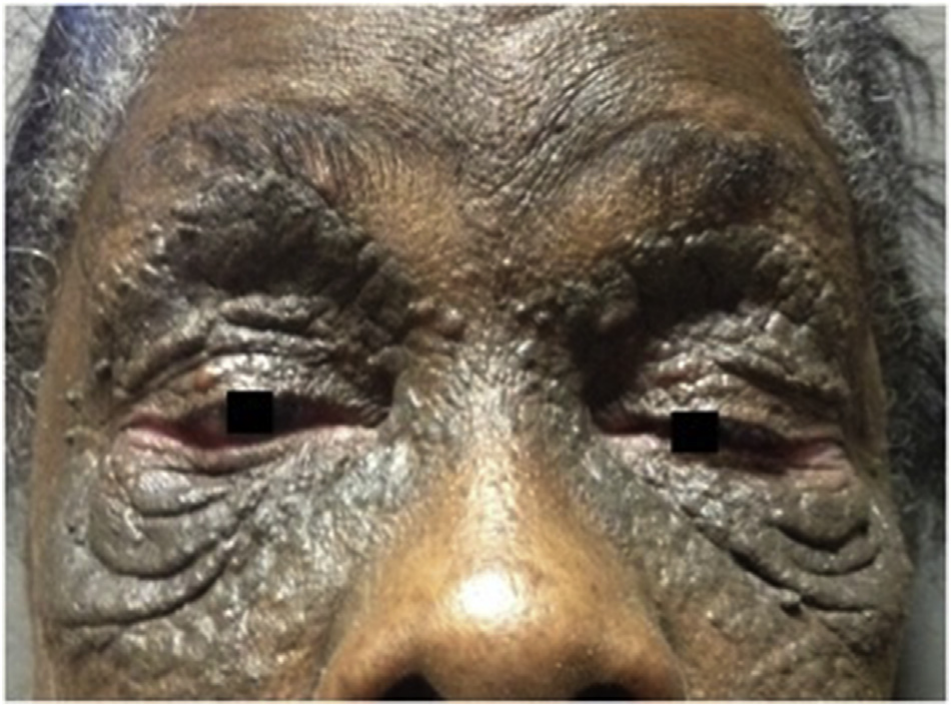
Before surgery Bilateral ptosis and ectropion of the lower eyelids.

**Fig. 2:**
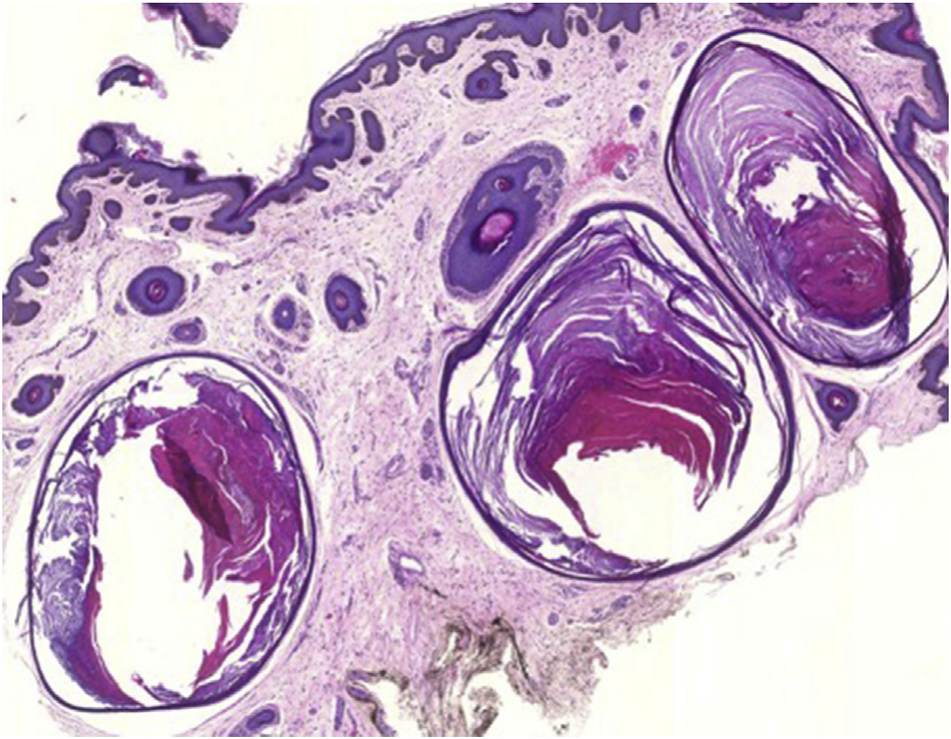
Histopathological analysis of the superficial skin resection (hematoxylin-eosin, original magnification x 20) Numerous cysts filled with keratin in the dermis.

**Fig. 3:**
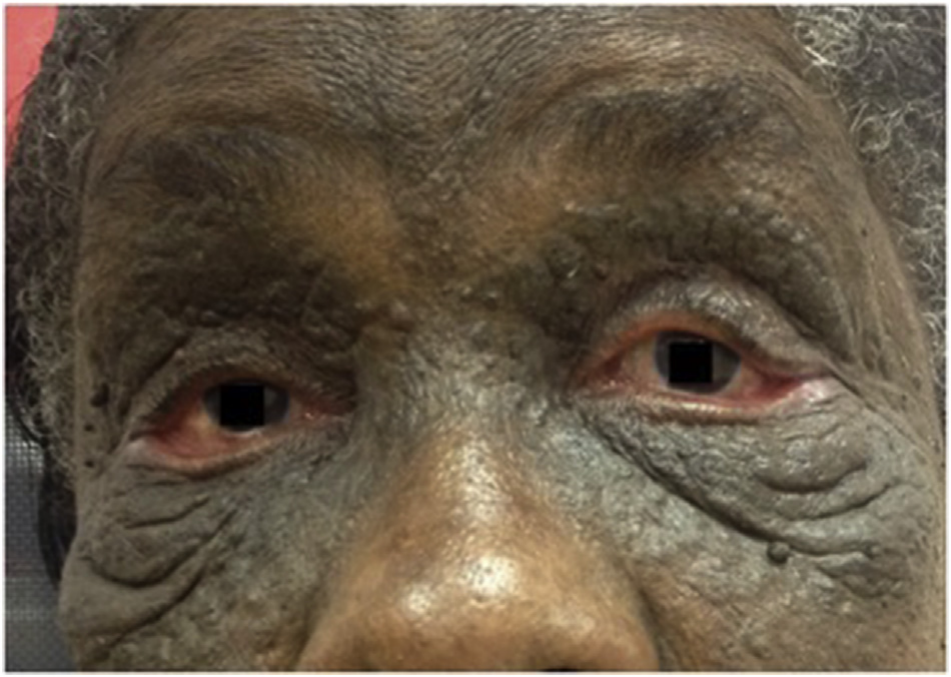
One month after surgery Ptosis was corrected.

A 69-year-old African Caribbean woman presented with a complaint of bilateral upper visual field defect in the setting of long-standing peri-orbital cutaneous lesions. She had a history of kidney graft secondary to terminal polycystic kidney disease. She had no history of trauma or periocular inflammation to the eye or the head. The cutaneous lesions appeared around the age of 35 with extensive progression. They were keratotic and hyperpigmented with multiple papules. Ophthalmologic examination showed bilateral ptosis and ectropion of the lower eyelids with bilateral punctuated superficial keratitis and inferior corneal pannus (Fig. 1). Pupils were equal and symmetric and there was no relative afferent pupillary defect. Eye motility was full. Interpalpebral fissure was 2mm on the left and 4mm on the right side. The upper eyelid lift function measured 12mm. Full review of systems was unremarkable. Histopathological findings on skin biopsy typically show large, grouped, dilated follicular ostia filled with keratin and confirmed the diagnosis of nevus comedonicus (Fig. 2). It was an isolated nevus comedonicus because there were no associated lesions including ocular defects (coloboma, conjunctival dermolipoma, choristoma, corneal opacities, congenital cataract). Blepharoplasty was performed on both upper eyelids. The superficial skin resection was 18mm on the left and 15mm on the right side. Ptosis was corrected and patient very satisfied of the result after surgery (Fig. 3). Ectropion surgery was planned in future.

## 3 Discussion

Nevus comedonicus is one of the rarest forms of epidermal nevus. It is commonly present at birth or appears during childhood. It is usually asymptomatic and unilateral with a predilection for the face, neck, chest.^1^ This case is unusual as regards the late-onset and location of the nevus comedonicus on both eyelids. Very few cases of nevus comedonicus with eyelid involvement were reported.^2^, ^3^, ^4^ The exclusive and bilateral location of the eyelids is exceptional because only three cases have been reported.^5^, ^6^, ^7^ Polat et al. reports a case in a 61-year-old man with bladder cancer. The lesions were localized to the 2 upper eyelids, had been evolving for about 6 months and had regressed with the application of tretinoin.^7^ The bilateral lesions were not like the clean-cut linear configuration from classic presentation of nevus comedonicus. The patient of this article is the first reported example of symptomatic bilateral nevus comedonicus of the eyelids with bilateral ptosis and ectropion. Differential diagnoses were eyelid dermatochalasis and floppy eyelid syndrome but quickly removed with this cutaneous lesion. MADISH (Metabolizing Acquired Dioxin Induced Skin Hamartoma) was also excluded because toxic exposure was not found. The weight of the eyelid lesions resulted in bilateral descent of the upper and lower eyelids. It is benign disease involving only superficial dermis without eyelids muscular defect. There are no similar cases in literature, so the long-term evolution is not predictable. The immediate post-operative scar showed no inflammatory reaction.

## 4 Conclusion

This case is unusual as regards the late-onset and location of the nevus comedonicus on both eyelids. Comedonicus nevus are benign lesions limited to the superficial dermis and require surgery for cosmetic and functional improvement, if there are upper visual field defect or corneal exposure.

## Patient consent

I certify receiving and having archived the written consentement of Mme X, who has Nevus comedonicus palpebral about potential medical press publication of her clinical observation. This report does not contain any personal identifying information.

## Authorship

All authors attest that they meet the current ICMJE criteria for Authorship.

## Funding

We have no financial interest or conflicting relationship concerning the subject of this paper.

## Declaration of competing interest

The following authors have no financial disclosures: Adam T, Hage R, Ahomadegbe C, Molinié V, Baubion E, Merle H.
